# Patient-centered disease management (PCDM) for heart failure: study protocol for a randomised controlled trial

**DOI:** 10.1186/1471-2261-13-49

**Published:** 2013-07-09

**Authors:** David B Bekelman, Mary E Plomondon, Mark D Sullivan, Karin Nelson, Brack Hattler, Connor McBryde, Kenneth G Lehmann, Jonathan Potfay, Paul Heidenreich, John S Rumsfeld

**Affiliations:** 1 Department of Veterans Affairs, Eastern Colorado Health Care System, Research (151), 1055 Clermont Street, 80220 Denver, CO, USA; 2 Department of Medicine, University of Colorado School of Medicine at the Anschutz Medical Campus, Aurora, CO, USA; 3 Department of Veterans Affairs, Eastern Colorado Health Care System Cardiology (111B), 1055 Clermont Street, Research (151), 80220 Denver, CO, USA; 4 Department of Psychiatry and Behavioral Sciences, University of Washington, Box 356560, 98195 Seattle, WA, USA; 5 VA Puget Sound Health Care System, 1660 South Columbian Way, 98108 Seattle, WA, USA; 6 Northwest HSR&D Center of Excellence, Seattle, WA, USA; 7 Department of Medicine, University of Washington, Seattle, WA, USA; 8 Department of Veterans Affairs, Eastern Colorado Health Care System, Ambulatory Care 11-B, 1055 Clermont Street, 80220 Denver, CO, USA; 9 Richmond VA Medical Center, 1201 Broad Rock Blvd, 23249 Richmond, VA, USA; 10 VA Palo Alto Health Care System Cardiology (111C), 3801 Miranda Avenue, 94304 Palo Alto, CA, USA

**Keywords:** Heart failure, Clinical trial, Patient reported outcomes, Quality of life, Health status

## Abstract

**Background:**

Chronic heart failure (HF) disease management programs have reported inconsistent results and have not included comorbid depression management or specifically focused on improving patient-reported outcomes. The Patient Centered Disease Management (PCDM) trial was designed to test the effectiveness of collaborative care disease management in improving health status (symptoms, functioning, and quality of life) in patients with HF who reported poor HF-specific health status.

**Methods/design:**

Patients with a HF diagnosis at four VA Medical Centers were identified through population-based sampling. Patients with a Kansas City Cardiomyopathy Questionnaire (KCCQ, a measure of HF-specific health status) score of < 60 (heavy symptom burden and impaired quality of life) were invited to enroll in the PCDM trial. Enrolled patients were randomized to receive usual care or the PCDM intervention, which included: (1) collaborative care management by VA clinicians including a nurse, cardiologist, internist, and psychiatrist, who worked with patients and their primary care providers to provide guideline-concordant care management, (2) home telemonitoring and guided patient self-management support, and (3) screening and treatment for comorbid depression. The primary study outcome is change in overall KCCQ score. Secondary outcomes include depression, medication adherence, guideline-based care, hospitalizations, and mortality.

**Discussion:**

The PCDM trial builds on previous studies of HF disease management by prioritizing patient health status, implementing a collaborative care model of health care delivery, and addressing depression, a key barrier to optimal disease management. The study has been designed as an ‘effectiveness trial’ to support broader implementation in the healthcare system if it is successful.

**Trial registration:**

Unique identifier: NCT00461513

## Background

Despite advances in chronic heart failure (HF) therapies, HF is a leading cause of disability, hospitalization, and death in the United States [1]. Moreover, HF has a major impact on patients’ health status, including their symptom burden (e.g. dyspnea), functional status, and health-related quality of life. However, few HF interventions have specifically targeted these critical patient-centered outcomes. In addition, diminished patient health status is predictive of HF hospitalization, mortality, and resource utilizations, yet health status has not been used to screen for patients to target disease management interventions [2].

While disease management has been variously defined and implemented, some previous studies have reported that HF disease management can reduce rates of hospitalization, and a few have demonstrated reductions in mortality, reductions in cost, or improvements in quality of life. However, many of these studies have been small, single-center trials of short duration, and the association between disease management and improved outcomes has been inconsistent [3-6]. Many HF disease-management studies to date have relied solely on nurse case management rather than multidisciplinary collaborative care, have not leveraged health information technology, and/or have had a limited focus on patient self-care. Most have not included screening and treatment of comorbid depression, or specifically targeted improvement in patient health status as the primary outcome [7]. The Patient-Centered Disease Management (PCDM) trial was designed to address these limitations.

The PCDM trial is evaluating the effectiveness of a collaborative care intervention with telemonitoring and evidence-based HF and depression management in an at-risk population of HF patients with diminished health status. All HF patients from participating centers were identified using existing VA electronic health record databases. The primary outcome of the PCDM trial is 1-year change in patient-reported HF health status, measured using the Kansas City Cardiomyopathy Questionnaire (KCCQ), comparing patients randomized to usual care versus the PCDM intervention. Secondary outcomes include depression, adherence to guideline-based therapies, hospitalizations, and mortality.

## Methods/design

### Study design overview

The primary objective of the PCDM trial is to determine whether a collaborative care HF disease management intervention, including depression assessment and treatment, improves patient-reported health status between baseline and 12 months, as measured by the KCCQ. The study was funded by the United State Department of Veterans Affairs (VA) and conducted in four VA medical centers across the U.S. The VA’s comprehensive electronic medical record was used to identify potential participants for the study. All patients with HF at the four sites were screened with the KCCQ. Eligible patients with diminished heart failure-specific health status (KCCQ summary score <60) were invited to an enrollment visit. Patients provided informed consent, were randomized to the PCDM intervention or usual care, and were followed for a 12-month period. The study was approved by the Institutional Review Boards at each of the study sites.

### Conceptual framework

The conceptual framework for the PCDM intervention highlights HF as a chronic illness often accompanied by co-occurring depression, both of which are prime candidates for disease management approaches, and the importance of health information technology, self-management, and health status outcomes. The Chronic Care Model [8] combines patient self-management support with health system change to achieve productive interactions between a “proactive health care team” and an “activated patient”, producing improved functional and clinical outcomes. The PCDM intervention uses collaborative care as the primary health system change. Collaborative care is the use of multidisciplinary teams to deliver evidence-based treatment to a defined population of patients with chronic illness [9]. The intervention aims to activate patients by promoting self-management of HF and depression through home telemonitoring and education, and when indicated, through integrating depression care into chronic illness care [10]. While telemonitoring in isolation is not effective in improving mortality or hospitalization, [11] telemonitoring might be a useful adjunct to the collaborative care model of health care delivery.

### Setting and population

**Figure 1:**
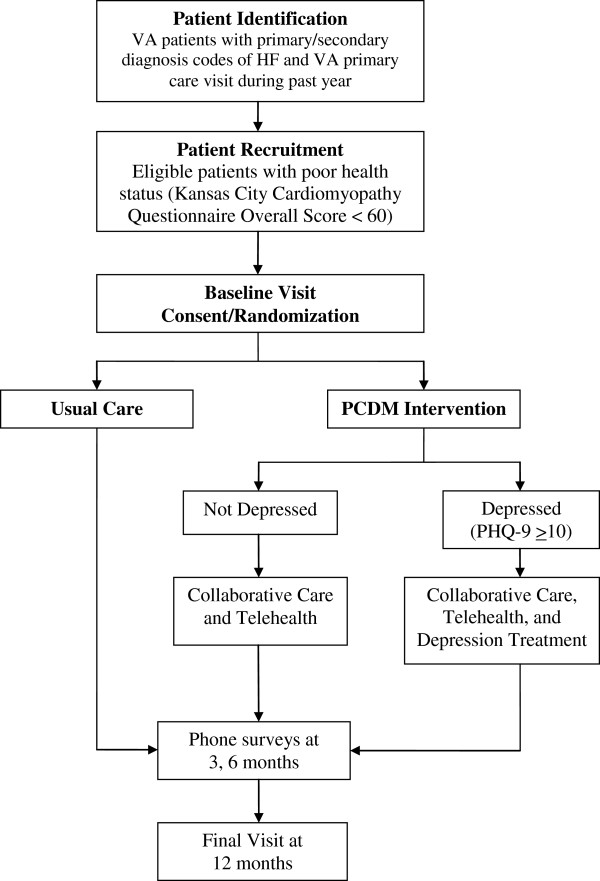
Patient-centered disease management (PCDM) for heart failure trial study population flow.

The study is being conducted at the Denver, Palo Alto, Richmond, and Seattle VA Medical Centers and their affiliated community-based outpatient clinics. All patients with an assigned primary care provider and at least one primary care provider visit during the prior 12 months with a HF diagnosis code in the VA electronic health record were screened for eligibility to participate (see Figure 1 for study population flow).

The study was purposefully designed to target patients with a diagnosis of HF in the VA electronic health record, irrespective of type of heart failure (e.g. with or without preserved left ventricular systolic function). Accordingly, the diagnosis of HF was defined as meeting any one of the following five criteria: (1) A primary inpatient hospital discharge diagnosis of HF (ICD-9 code 428.XX); (2) ≥2 secondary inpatient hospital discharge diagnoses of HF (ICD-9 codes 428.XX) and a primary inpatient hospital discharge diagnosis related to heart disease (ICD-9 codes 410.XX, 412.XX, 413.XX, 414.XX); (3) ≥3 secondary inpatient hospital discharge diagnosis codes related to HF; (4) ≥2 outpatient visit diagnoses of HF, excluding emergency department visits; (5) ≥2 secondary inpatient hospital discharge diagnoses of HF and ≥ 1 outpatient HF diagnosis. This case-finding methodology has been previously described [12]. The primary care provider of each patient was contacted by mail to inform them of the PCDM trial and to allow them to withdraw their patient from further consideration if they deemed the patient to be a poor candidate for study participation.

### Recruitment and study subjects

Following the process of patient identification described above, all remaining patients were mailed a study informational sheet, a decline-to-participate postcard, and a KCCQ questionnaire. Individuals not responding within two weeks received a reminder letter, followed by a phone call if necessary. All returned KCCQ questionnaires were scored, and the individuals scoring < 60 were invited to an enrollment visit if they did not meet any of the exclusion criteria. The exclusion criteria included: (1) cognitive or psychiatric impairment that precludes completion of questionnaires; (2) current residence in a nursing home; (3) irreversible, non-cardiac medical conditions (e.g. metastatic cancer) likely to affect 6-month survival or ability to execute the study protocol; (4) absence of a telephone line in the home; (5) inability to read English; (6) prior heart transplantation; (7) Alcohol Use Disorders Identification Test (AUDIT-C) score ≥ 7 [13].

### Randomization

At the enrollment visit, patients provided informed consent, completed baseline survey measures, and were then randomized. The random allocation sequence was computer generated (SAS version 9.1 procedure PLAN) using a uniform fixed randomization design with an allocation ratio of 1:1 and stratification by site.

### Intervention

#### Collaborative care

The collaborative care team at each site consisted of a local primary care provider, cardiologist, psychiatrist, and nurse coordinator (registered nurse). The team assessed each intervention patient through a review of the VA electronic health record and baseline depression scores from the Patient Health Questionnaire-9 (PHQ-9). The team recommended care changes, as indicated, for a given patient in accordance with the ACC/AHA Guidelines for the Management of Chronic Heart Failure [14] and depression care and telemonitoring data as described below. Collaborative care team recommendations were entered into a progress note in the electronic medical record for review and co-signature by the patient’s primary care provider. Unsigned orders were placed for primary care providers to review and sign at their discretion. This methodology was successfully used within the VA in a study of collaborative care for patients with angina [15].

#### Screening and treatment of depression

Patients were screened for depression at the initial baseline visit using the PHQ-9. Patients who scored ≥ 10 who were randomized to the intervention were entered into the depression care component of the intervention, adapted from a successful collaborative depression care intervention [16]. This included: (1) up to 11 sessions behavioral activation and antidepressant management provided by the nurse coordinator and supervised by the team psychiatrist, with approximately half of visits planned to be by phone and half in person; (2) a depression educational video; (3) depression assessment and self-management education via telemonitoring. The nurse coordinator from each site participated in a two-day training on depression and behavioral activation by the lead study psychiatrist and four weekly follow up calls with the lead psychiatrist. Thereafter, the nurse was supervised by the site psychiatrist with as needed calls with the lead psychiatrist.

#### Telehealth: telemonitoring and patient self-care support

Intervention patients received daily telemonitoring using equipment that has modules for both HF and depression. The system collected blood pressure, pulse, weight, and self-reported symptoms. Patients were asked a pre-programmed series of questions such as, “Are you more short of breath than usual today?”; “Do you have any new or any more swelling than usual in your feet or ankles today?” Patients in the depression care component of the intervention were asked questions about their mood and behavior. Follow-up questions may be generated using branching-chain logic. For instance, a patient responding that he/she is experiencing shortness of breath is then asked to rate the severity. Thresholds are set for notification of the nurse coordinator or for the patient to call the nurse coordinator. The nurse coordinator reviewed the telemonitoring data and could then either provide counseling to the patient or present the patient to the collaborative care team for recommendations.

The self-care programs included medication reminders to promote adherence, education about HF and depression, medication monitoring, and dietary advice. Patients were also taught signs and symptoms to report and ways to manage HF and cardiovascular risk factors such as proper technique and importance of daily self-weighing, adherence to a low sodium diet and medication regimens, and recognition of early signs and symptoms of HF decompensation.

### Usual care

Patients randomized to the usual care arm continued to receive care from their regular medical providers. This care was fully at the discretion of the patient’s regular clinicians, and thus may or may not have included cardiology or mental health clinic care in addition to primary care. Telemonitoring was offered to usual care patients if they were referred for home telemonitoring by any of their providers. However, the telemonitoring data was handled as part of usual care by VA telemonitoring nurses at each facility, rather than being provided to the collaborative care team in the PCDM intervention. At the enrollment visit, patients randomized to the usual care arm were given information sheets that describe self-care for HF and were provided with a scale if needed. Primary care providers were notified if usual care patients screened positive for depressive symptoms based on the initial study surveys. From there, patients’ primary care clinicians assumed responsibility for depression care or referral, at their discretion.

### Outcome measures

The questionnaires and methods used to measure the study outcomes are listed in the Table 1 Baseline measures were collected in person, a research assistant (who was blinded to the randomization arm) collected 3 and 6 month follow-up study outcome measures by phone or in person, and the nurse coordinator collected outcome measures at 12 months.

**Table 1:** Patient-centered disease management (PCDM) for heart failure trial study outcomes

**Domain**	**Measure**	**Timing**			
		**Baseline**	**3-month**	**6-month**	**12-month**
**Primary Outcome**					
HF-specific Health Status	KCCQ^*^	X	X	X	X
**Secondary Outcomes**					
Depression	PHQ-9^†^	X	X	X	X
	SCL-20^‡^	X			X
Medication Adherence	PDC^§^	X	X	X	X
Guideline-Based Care	Criteria mapping	X			X
Hospitalizations, mortality	Medical Record Review, Self- or Surrogate Report				X

^*^ Kansas City Cardiomyopathy questionnaire.

† Patient health questionnaire-9.

^‡^ Hopkins symptom checklist-20.

^§^ Proportion of days covered.

The primary outcome, HF-specific health status, was measured using the KCCQ. The KCCQ is valid, reliable, sensitive to clinical change, and predicts hospitalization and mortality [2,17,18]. For the secondary outcomes, depression was measured using the PHQ-9 and the Hopkins Symptom Checklist-20 (SCL-20). The PHQ-9 is a valid and reliable instrument that provides a continuous measure of depressive symptoms [19]. The Hopkins Symptom Checklist-20 (SCL-20) is also a valid and reliable measure of depression symptoms [20]. To examine medication adherence, the proportion of days covered (PDC) will be calculated for each patient using established methods, based on the total number of days supplied for filled prescriptions over the observation time interval [21]. Primary medications to be evaluated will be angiotensin converting enzyme inhibitors, angiotensin II receptor blockers, beta-blockers, aldosterone antagonists, statins, digoxin, diuretics, and antidepressants. For a given patient, the proportion of guideline-concordant care will be determined using criteria mapping [15,22]. The guideline-concordant care analysis will be restricted to those patients who have a guideline indication for a medication class, such as beta-blockers for HF with left ventricular systolic dysfunction. Hospitalizations and mortality will be assessed through VA databases, supplemented by patient self-report. Vital status will also be ascertained via the VA Vital Status File which has a sensitivity of 98.3% and specificity of 99.8% compared to the National Death Index [23].

### Sample size and statistical power

The primary hypothesis of this study is that HF patients receiving the PCDM intervention will have improved health status over a 12-month follow-up period compared with those receiving usual care. The minimal clinically meaningful difference in KCCQ summary scores is 5 points, [18] and the standard deviation of change in KCCQ scores was estimated to be between 15 and 20 based on previous work [17,18]. Using a type I error rate (α) of 0.05, and an attrition rate of 30%, we originally estimated enrollment of 600 patients would have >80% power to detect a difference in KCCQ scores of ≥ 5 points assuming a standard deviation of 18. The data to date showed less than 20% attrition and a KCCQ standard deviation of 15 points. With the final enrollment of 392 patients, we will have over 80% power to detect a 5 point difference in KCCQ score.

### Analyses

All primary and secondary analyses will be conducted using an intention-to-treat approach. Patients will be analyzed in the arm to which they were randomized irrespective of treatment adherence. The baseline characteristics of patients will be summarized by study group and examined for differences. To examine the primary outcome, change in HF-specific health status as measured by the KCCQ, a likelihood-based random effects model using all available data will be implemented [24]. We will examine the magnitude and patterns of missing data to check the sensitivity of the model assumptions. Model parameters will be estimated using the SAS procedure MIXED (SAS Institute, Cary, NC). Most of the secondary study outcomes are continuous and will be analyzed using a similar mixed modeling approach. Rates of all-cause hospitalization and mortality and the proportion of patients in each study arm receiving guideline-concordant care at 12-months will be compared between study arms using a Chi-Square test. To examine medication adherence, the PDC for each study group will be compared as a continuous measure (t-test) and as the proportion of patients achieving a PDC > 0.80 (Chi-Square), a cut-off used in multiple medication adherence studies [25].

### Limitations/considerations

It is possible that a given primary care provider will have patients in both the intervention and control arms and this will lead to contamination. However, study patients represent a very small number of the patient panels of a given primary care provider, and thus the potential for contamination is low. The intervention is multimodal, and we may not know the most important components of the intervention if it is successful. However, as unimodal interventions have not been very successful in changing health care delivery and health status outcomes, multimodal interventions are necessary [26]. Process monitoring of the intervention through assessment of collaborative care team recommendations should provide insights about important intervention components.

As in many HF studies, we may inadvertently enroll some patients who do not have HF by criteria such as Framingham, [14] but this group will be of inherent interest from the health care system perspective. The rationale for this inclusive approach is that the study was designed as an effectiveness trial to support broader implementation if the intervention is successful. We feel it is important to consider all patients who have a HF diagnosis in the medical record and have low HF-specific health status, above and beyond any physiologic/anatomic measures (e.g. echo findings) for which is there is no gold-standard for the diagnosis of HF. Similarly, we believe it would be wrong to only include patients with systolic dysfunction, since patients with HF and preserved systolic function are prevalent and also have diminished health status and poor prognosis [27]. Among enrolled patients, we will carefully collect and classify medical history and tests to be able to report the proportions of patients with and without systolic dysfunction and the etiology of HF.

## Discussion

The PCDM trial is unique because it uses a patient-centered measure for study entry and as the primary outcome. It employs population-based case finding methods to target HF patients with diminished self-reported health status who are at high risk and who may benefit most from disease management. The PCDM intervention uses collaborative care to address both HF and comorbid depression and leverages health information technology to provide optimal disease management. The trial builds on previous studies of HF disease management by prioritizing patient health status, implementing a collaborative care model of health care delivery, and addressing depression, a key barrier to optimal disease management. The study has been designed as an ‘effectiveness trial’ to support broader implementation in the healthcare system if it is successful.

## Abbreviations

HF: Chronic heart failure; PCDM: Patient-centered disease management; KCCQ: Kansas city cardiomyopathy questionnaire; PHQ-9: Patient health questionnaire-9.

## Competing interests

The authors have no conflicts of interest to disclose.

## Authors’ contributions

JSR, MDS, and PH conceived the study. JSR and PH obtained funding for the study. The trial protocol was developed by HR, MEP, MDS, and PH. KN, BH, DBB, CM, KL, JP, and PH were site lead investigators. DBB and MDS oversaw the depression intervention. The first draft of this paper was written by DBB. All authors revised it critically for important intellectual content, and all authors read and approved the final manuscript.

## Pre-publication history

The pre-publication history for this paper can be accessed here:

http://www.biomedcentral.com/1471-2261/13/49/prepub
